# Supplementary material for: The small molecule regacin binds a conserved pocket in RegA to inhibit the expression of bacterial virulence factors
Source: bioRxiv. 2026 Sep 14:2026.09.11.750636. Preprint. [Version 2] doi: 10.64898/2026.09.11.750636 (PMC13596237; doi:10.64898/2026.09.11.750636)
Supplement: Supplementary file 1 [file NIHPP2026.09.11.750636v2-supplement-1.pdf]

bioRxiv preprint doi: https://doi.org/10.64898/2026.09.11.750636; this version posted September 14, 2026. The copyright holder for this preprint (which was not certified by peer review) is the author/funder, who has granted bioRxiv a license to display the preprint in perpetuity. It is made available under a CC-BY 4.0 International license.

Supplemental Figures

Table S1: Crystallographic Statistics for RegA and Regacin-RegA Structures

	RegA	Regacin-RegA
Wavelength	0.92010	0.92010
Resolution range	33.34 - 2.69 (2.74 - 2.69)	48.15 - 2.55 (2.63 - 2.55)
Space group	P 1 21 1	P 1 21 1
Unit cell a b c (Å)	68.293 98.569 86.170	68.154 98.842 86.255
Unit cell α β λ (°)	90.00 100.23 90.00	90.00 101.13 90.00
Total reflections	109358 (10033)	133789 (13848)
Unique reflections	31082 (2826)	42426 (4419)
Multiplicity	3.5 (3.6)	3.2 (3.1)
Completeness (%)	99.58 (99.08)	94.08 (99.45)
Mean I/sigma(I)	5.44 (0.81)	4.34 (0.82)
R-merge	0.1285 (1.248)	0.1092 (1.315)
R-meas	0.1522 (1.473)	0.1317 (1.585)
R-pim	0.08074 (0.7779)	0.07264 (0.8742)
CC1/2	0.994 (0.439)	0.991 (0.346)
Reflections used in refinement	31043 (2805)	34513 (3613)
Reflections used for R-free	1543 (118)	1689 (189)
R-work	0.2165 (0.3457)	0.2141 (0.3106)
R-free	0.2514 (0.3662)	0.2630 (0.3510)
Number of non-hydrogen atoms	8143	8231
macromolecules	8096	8120
ligands	1	35
solvent	46	76
Protein residues	1001	1004
RMS (bonds)	0.003	0.003
RMS (angles)	0.62	0.61
Ramachandran favored (%)	97.77	96.77
Ramachandran allowed (%)	2.23	3.12
Ramachandran outliers (%)	0	0.1
Average B-factor	75.05	60.53
macromolecules	75.12	60.51
ligands	68.4	77.71
solvent	62.2	54.92

bioRxiv preprint doi: https://doi.org/10.64898/2026.09.11.750636; this version posted September 14, 2026. The copyright holder for this preprint (which was not certified by peer review) is the author/funder, who has granted bioRxiv a license to display the preprint in perpetuity. It is made available under a CC-BY 4.0 International license.

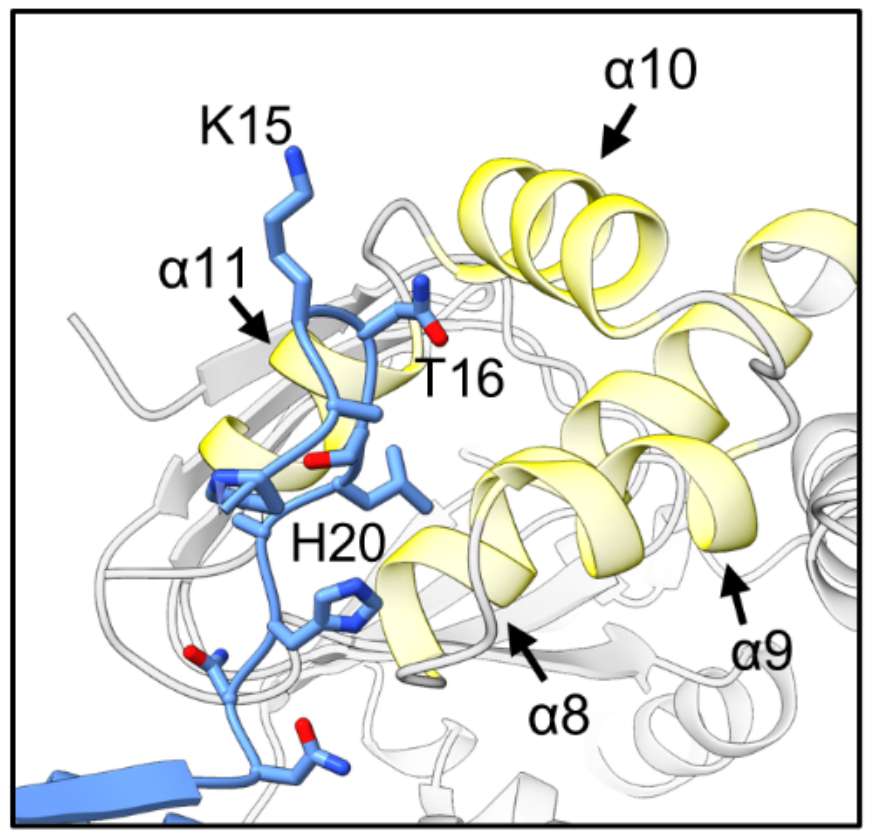

Figure S1: Regacin-RegA chain A amino-terminus (cornflower blue) interacting with the symmetry mate DBD helices α8, α9, α10, and α11 (yellow).

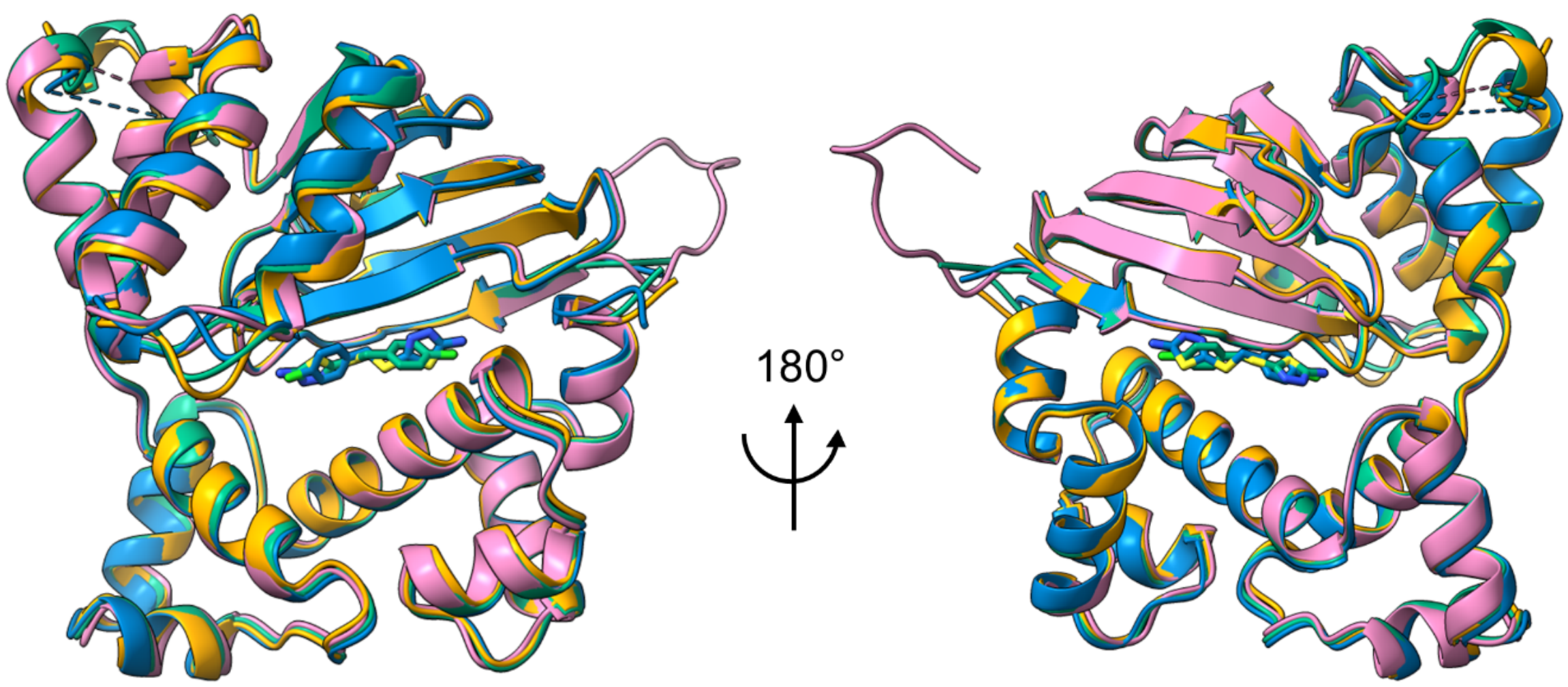

Figure S2: Each chain in the regacin-RegA asymmetric unit is aligned, and they are nearly superimposable. Chain A is shown in pink, B is orange, C is green and D is blue. The loop regions display variable conformations and are unresolved in some chains (chain breaks shown as dashed lines.) The regacins from chain C and D align very well within the pocket but are in different orientations.

bioRxiv preprint doi: https://doi.org/10.64898/2026.09.11.750636; this version posted September 14, 2026. The copyright holder for this preprint (which was not certified by peer review) is the author/funder, who has granted bioRxiv a license to display the preprint in perpetuity. It is made available under a CC-BY 4.0 International license.

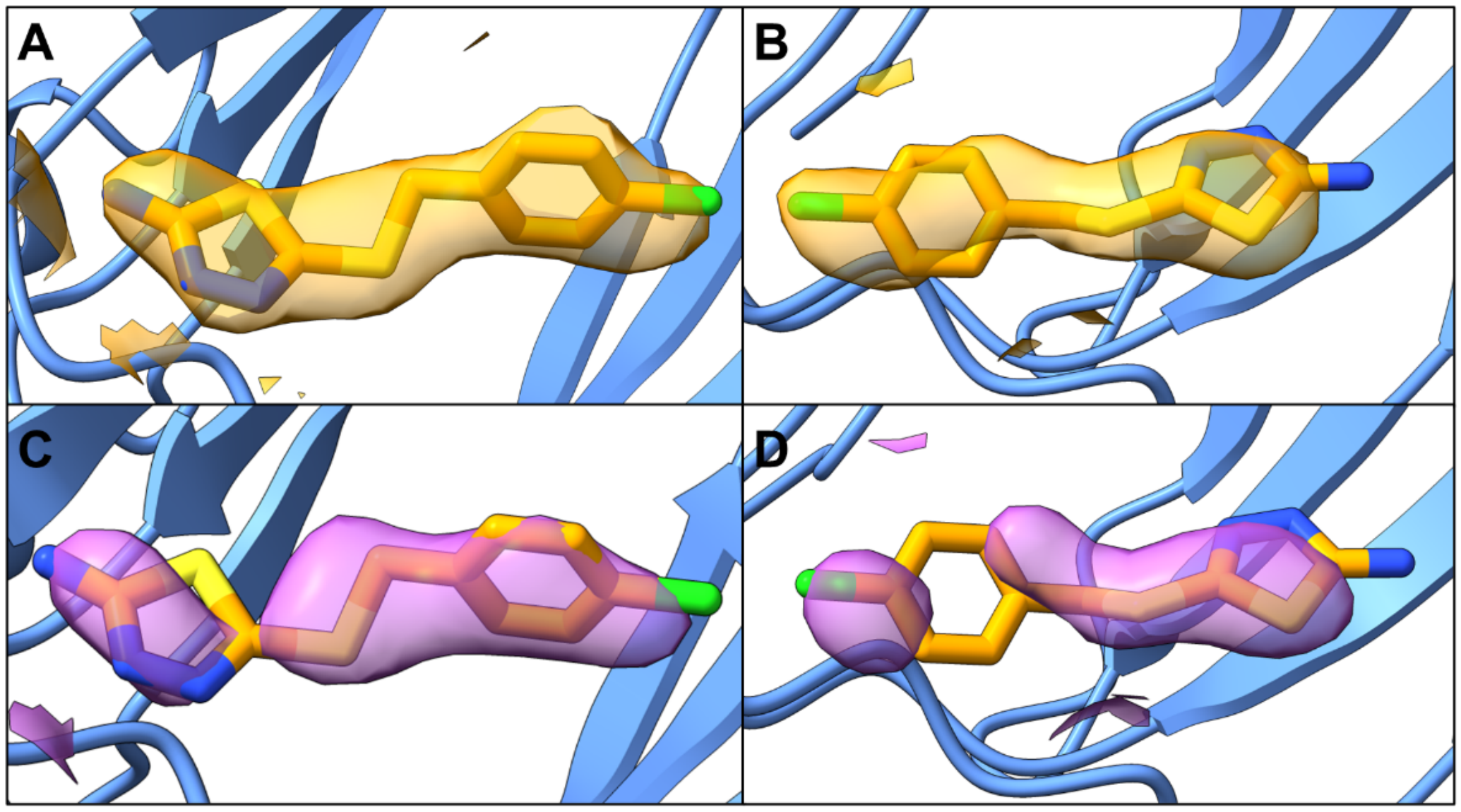

Figure S3: A) Regacin-RegA 2Fo-Fc electron density map (orange) contoured at 1 σ (RMSD) and displayed within a 2.5 Å radius of the regacin ligand bound in the pocket of chain B. **B)** Same map as in panel A, but showing the density around the regacin ligand in the pocket of chain D. **C)** Regacin-RegA composite-omi 2mFo-DFc map (purple) contoured at 1 σ (RMSD) and displayed within a 2.5 Å radius of the regacin ligand in the pocket of chain B. **D)** Same composite-omit map as in panel C, but for the regacin ligand bound in the pocket of chain D.
